# Clinical Significance of Serum Glutamine Level in Patients with Colorectal Cancer

**DOI:** 10.3390/nu11040898

**Published:** 2019-04-21

**Authors:** Hang Huong Ling, Yi-Ping Pan, Chung-Wei Fan, Wen-Ko Tseng, Jen-Seng Huang, Tsung-Han Wu, Wen-Chi Chou, Cheng-Hsu Wang, Kun-Yun Yeh, Pei-Hung Chang

**Affiliations:** 1 Division of Hemato-oncology, Department of Internal Medicine, Chang Gung Memorial Hospital, Keelung and Chang Gung University, College of Medicine, Keelung 204, Taiwan; xianfang87@gmail.com (H.H.L.); liting@cgmh.org.tw (J.-S.H.); u402026@gmail.com (T.-H.W.); chwang@cgmh.org.tw (C.-H.W.); yehtyng@gmail.com (K.-Y.Y.); 2 Department of Nutrition, Chang Gung Memorial Hospital, Keelung and Chang Gung University, College of Medicine, Keelung 204, Taiwan; pyngpyng@gmail.com; 3 Division of Colorectal Surgery, Chang Gung Memorial Hospital, Keelung and Chang Gung University, College of Medicine, Keelung 204, Taiwan; cwf2564@adm.cgmh.org.tw (C.-W.F.); tsangwen@cgmh.org.tw (W.-K.T.); 4 Division of Hemato-oncology, Department of Internal Medicine, Chang Gung Memorial Hospital, Linkou and Chang Gung University, College of Medicine, Taoyuan 333, Taiwan; wenchi3992@yahoo.com.tw

**Keywords:** colorectal cancer, serum glutamine level, survival, progression, inflammation

## Abstract

Limited studies have assessed the associations of pretreatment serum glutamine level with clinicopathological characteristics and prognosis of colorectal cancer (CRC) patients. This study focuses on clarifying the clinical significance of baseline serum glutamine level in CRC patients. We retrospectively examine 123 patients with newly diagnosed CRC between 2009 and 2011. The associations of pretreatment serum glutamine level with clinicopathological characteristics, proinflammatory cytokines, overall survival (OS), and progression-free survival (PFS) were analyzed. We executed univariate and multivariate analyses to assess the associations between serum glutamine level and clinicopathological variables able to predict survival. Low glutamine levels were associated with older age, advanced stage, decreased albumin levels, elevated carcinoembryonic antigen levels, higher C-reactive protein levels, higher modified Glasgow prognostic scores, and higher proinflammatory cytokine levels. Furthermore, patients with low glutamine levels had poorer OS and PFS than those with high glutamine levels (*p* < 0.001 for both). In multivariate analysis, pretreatment glutamine level independently predicted OS (*p* = 0.016) and PFS (*p* = 0.037) in CRC patients. Pretreatment serum glutamine level constitutes an independent prognostic marker to predict survival and progression in CRC patients.

## 1. Introduction

Colorectal cancer (CRC) is a prevalent and common cause of cancer deaths worldwide, with 832,000 deaths in 2015 [1,2]. Curative surgery remains the best chance of cure in CRC patients, while systemic targeted therapy and chemotherapy are the standards of care for disease recurrence or metastasis. However, some patients still have early progression and dismal outcome [3]. At present, the widely accepted prognostic factors for survival in CRC patients include tumor-node-metastasis (TNM) staging, histologic grade, tumor location, and carcinoembryonic antigen (CEA) level [4,5,6]. However, individual CRC patients demonstrate different survival times and recurrence. Therefore, the identification of novel biomarkers is necessary for better prognostic evaluation and treatment decisions in CRC patients.

Recent studies have utilized concentration changes in serum amino acid panels for the diagnosis and screening of prostate, ovarian, renal, and gastric cancers [7,8,9,10]. These amino acid panels, including glutamine, have also emerged as promising screening tools for the detection of CRC, although with limited validation and heterogeneous results [11,12,13,14]. Colorectal carcinogenesis is also associated with metabolic changes which result in a large number of metabolites [15]. Glutamine is the most abundant amino acid, accounting for over 20% of the amino acids in plasma and 40% in muscle [16]. It is maintained in circulation at a relatively constant level of 0.6–0.9 mmol/L [17]. Besides its source via diet ingestion, glutamine is considered a non-essential amino acid, because it can also be synthesized endogenously by human skeletal muscles, lungs, or adipose tissue [18]. Glutamine may become a conditionally essential amino acid during the rapid growth of proliferating cells, certain cancer cells, or under stress condition. In cancer cells, glutamine plays versatile roles in bioenergetics, macromolecular synthesis, and redox homeostasis to promote cell proliferation and survival [19]. Avid glutamine consumption and metabolism of cancer cells have become fields of interest in recent years in an effort to discover new therapeutic targets in cancer patients [20]. The decreased concentration of glutamine in serum may be associated with a higher demand by proliferating cancer cells [21]. Recent metabolomic studies have reported significantly decreased serum glutamine levels in CRC compared to those in healthy individuals [12,13,14]. Researchers have mostly focused on the diagnostic value of glutamine in CRC and limited studies have investigated the prognostic and predictive values of glutamine [14,22]. Accordingly, we aimed to clarify the relationships between pretreatment serum glutamine level with clinicopathological features and prognosis in patients with CRC.

## 2. Materials and Methods

### 2.1. Participants

We retrospectively enrolled 164 patients visiting the Keelung branch of Taiwan’s Chang Gung Memorial Hospital; they received a new diagnosis of CRC. The study recruitment period was from 2009 to 2011. For all patients, data concerning the following were obtained and analyzed: Age, sex, body mass index, TNM stage, tumor location, histologic differentiation, CEA level, albumin level, C-reactive protein (CRP) level, tumor necrosis factor (TNF)-α level, interleukin (IL)-1β level, IL-6 level, overall survival (OS), and progression-free survival (PFS). We specified OS and PFS as the time between the diagnosis date and any-cause mortality and as the time between the diagnosis date and first evidence of progression, respectively. We followed up the patients until death or 30 June 2016. Moreover, we executed tumor classification in conformity with the American Joint Committee on Cancer Staging System 7th edition (AJCC 7th edition) on the basis of computed tomography, physical examination, chest radiography, and routine laboratory tests. Modified Glasgow prognostic scores (mGPSs) were obtained using albumin and CRP levels. When both levels were abnormal (CRP and albumin levels >10 mg/L and <3.5 g/dL, respectively), a score of 2 was given; when the CRP level was >10 mg/L, but the albumin level was normal, the score was 1; and when the CRP level was <10 mg/L regardless of albumin level, the score was 0 [23]. Our institute’s CRC committee reviewed all the enrolled patients’ pathological diagnoses. This committee comprised three medical oncologists, three colorectal surgeons, two pathologists, and two radiation oncologists. We excluded patients with recurrent cancer, another concomitant active cancer, any active infection, or inflammatory diseases. Forty-one patients were excluded because their biochemistry data were incomplete (23 patients) or glutamine data were missing (18 patients). After the exclusions, data of 123 patients were analyzed. The ethical principles of the Declaration of Helsinki were followed in this study, and we obtained ratification for the executed study from the hospital’s institutional review board (201204587B0). All patients provided written informed consent before being enrolled.

### 2.2. Measurement of Serum Glutamine Levels

We collected pretreatment blood samples for glutamine analysis in heparinized tubes and subjected them to 15-min centrifugation executed at 1500 rpm. Next, we stored the acquired serum samples at −80 °C until they were analyzed. Serum glutamine concentration was measured using an Enzy ChromTM Glutamine Assay Kit (EGLN-100, Bio Assay Systems, Hayward, CA, USA). We thawed the acquired samples once only, and we executed measurements in triplicate.

### 2.3. Measurement of Proinflammatory Cytokine Levels

We collected blood samples from the enrolled patients prior to their treatment, promptly followed by subjecting them to 15-min centrifugation executed at 500× *g*. Prior to processing, we stored all serum samples at −80 °C. The DuoSet ELISA Development kit (R&D Systems, Minneapolis, MN, USA) was employed to determine the serum IL-6, TNF-α, and IL-1β levels; the kit was executed in conformity with the manufacturer’s instructions. We obtained the final levels through detection using a luminescence counter system (Packard Instrument Company Downers Grove, IL, USA). Similarly, we thawed the derived samples once only, and we executed measurements in triplicate.

### 2.4. Statistical Analysis

Statistical analyses were performed using IBM SPSS Statistics for Windows, version 19.0 (IBM Corp., Armonk, NY, USA). The optimal serum glutamine cutoff value was determined by the Youden’s index based on the receiver operating characteristic curve analysis. The relationships between pretreatment glutamine levels and clinicopathological characteristics were assessed using Pearson’s chi-square tests. By applying the Kaplan–Meier method, we derived cumulative survival curves; in addition, we determined intergroup differences through the execution of log-rank tests. We assessed the clinical variables by employing a univariate Cox proportional hazards model, with statistically significant variables further assessed by constructing a multivariate Cox model through the forward stepwise approach. Furthermore, the correlations between glutamine level and the levels of proinflammatory cytokines were examined using independent-samples t-tests. We specified *p* < 0.05 (two-tailed) as signifying statistical significance.

## 3. Results

### 3.1. Patient Characteristics

The clinicopathological features of the 123 eligible patients are summarized in Table 1. The male:female ratio among the patients was 78:45 (63.4%:36.6%), and the median (range) age observed at diagnosis was 67 (18–94) years. Some previous pieces of literature have demonstrated “U-shaped” relationship between BMI and long-term survival outcomes, in which both underweight and obese patients portrayed significantly increased mortality compared to the normal or overweight group [24,25,26]. Considering the heterogeneity of BMI among geographic regions and ethnicities, we categorized the patients into four groups according to the BMI classification of Department of Health in Taiwan, which consisted of underweight (BMI < 18.5 kg/m^2^), normal (18.5 to 23.9 kg/m^2^), overweight (24 to 26.9 kg/m^2^) and obese (≥27 kg/m^2^) groups [27]. 14 (11.4%) patients were underweight, 61 (49.6%) patients had a normal BMI, 31 (25.2%) patients were overweight, and 17 (13.8%) patients were obese. According to AJCC 7th edition criteria, 27 patients (22.0%) had stage I, 33 (26.8%) had stage II, 41 (33.3%) had stage III, and 22 (17.9%) had stage IV disease. Among the stage IV patients, all of them were synchronous metastases at the time of diagnosis. 73 (59.3%) of stage IV patients had more than one metastatic site. Liver constituted the most common metastatic site (68.1%), followed by lung (31.8%), non-regional lymph nodes (27.3%), and peritoneal metastasis (22.7%). The colon was the most common tumor location (83 cases; 67.5%), with the location being the rectum in all other cases (40 cases; 32.5%). Histology was assessed using the World Health Organization histologic grading of adenocarcinoma, and in 39 cases, the tumors were well differentiated (31.7%); in 76 cases, moderately differentiated (61.8%); and in 8 cases, poorly differentiated (6.5%). 

The mean serum glutamine concentration was 95 ± 121 ng/μL. We evaluated the impact of serum glutamine level on clinicopathological features and survival. Scholars have not reached consensus regarding the most appropriate serum glutamine concentration cutoff, because the studies conducted have had inherent enrollment differences regarding disease variation, ethnicity, and measurement methodology. According to the ROC analysis in the present study, the optimal glutamine cutoff value based on the OS was 51.75 ng/μL, with an area under the curve of 0.279 (95% confidence interval (CI): 0.189–0.368; *p* < 0.001). We thus arbitrarily stratified patients into low glutamine (<52 ng/μL, *n* = 72) and high glutamine (≥52 ng/μL, *n* = 51) groups. The relationships between pretreatment glutamine levels and the clinicopathological characteristics of CRC patients are demonstrated in Table 1. We noted a significant association between lower pretreatment glutamine level and older age (*p* < 0.001), advanced stage (*p* = 0.019), higher CEA level (*p* = 0.001), lower albumin level (*p* = 0.005), higher CRP level (*p* = 0.003), and higher mGPS (*p* = 0.004).

### 3.2. Survival Analysis

**Figure 1:**
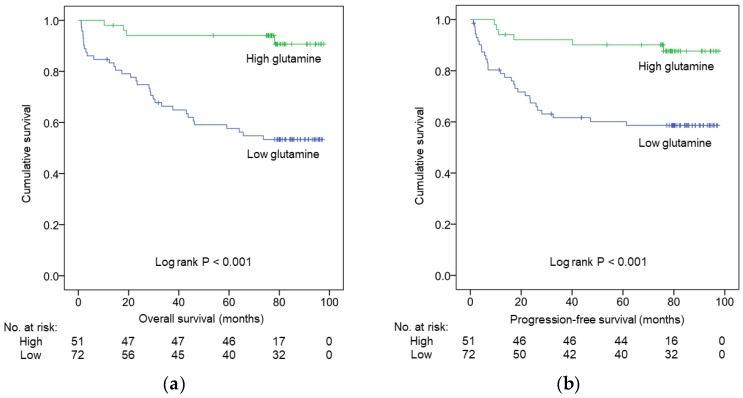
Kaplan–Meier survival curves showing (**a**) overall survival (OS) and (**b**) progression-free survival (PFS) of colorectal cancer (CRC) patients according to pretreatment serum glutamine levels. P-values were determined using log-rank test.

Our study’s median (range) duration of follow-up was determined to be 78.5 (1.1–97.7) months. At the end of the analysis, the disease of 35 (28.5%) patients had progressed and 37 (30.1%) patients had died. According to Kaplan–Meier analysis, patients with low glutamine levels had significantly poorer OS and PFS (*p* < 0.001 for both, Figure 1a,b, respectively) than those exhibiting high glutamine levels. Similarly, we determined patients with low glutamine levels to exhibit lower 5-year OS (57.7% vs. 94.0%) and 5-year PFS (60.1% vs. 90.1%) rates than did those with high glutamine levels.

### 3.3. Prognostic Factors Influencing OS and PFS

In univariate analyses, BM I< 18.5 kg/m^2^; stage; and levels of CEA, albumin, CRP, mGPS, and glutamine were significantly associated with OS (Table 2) and PFS (Table 3). In the multivariate Cox regression model, advanced stage (hazard ratio (HR) = 3.803; 95% CI 2.382–6.073; *p* < 0.001), low albumin level (HR = 0.401; 95% CI 0.204–0.786; *p* = 0.008), and low glutamine level (HR = 0.270; 95% CI 0.093–0.787; *p* = 0.016) were found to independently and significantly predict OS. Moreover, advanced stage (HR = 7.305; 95% CI 3.996-13.355; *p* < 0.001), low BMI (HR = 0.231; 95% CI 0.085–0.631; *p* = 0.004), and low glutamine levels (HR = 0.367; 95% CI 0.143–0.941; *p* = 0.037) remained significant prognostic factors for PFS.

### 3.4. Correlation between Serum Glutamine and Proinflammatory Cytokine Levels

We further evaluated the relationships between pretreatment serum glutamine and proinflammatory cytokine levels (Table 4). Lower glutamine levels were significantly associated with higher levels of proinflammatory cytokines, such as IL-1β (*p* = 0.030) and IL-6 (*p* = 0.041). A trend toward an elevated TNF-α level (*p* = 0.104) was also noted in the low glutamine group.

## 4. Discussion

Innovations in cancer metabolomics in recent years has led to the identification of abundant biomarkers in various types of cancers, with the aim of better diagnostic, prognostic, and predictive values [28]. Aside from genomic and proteomic alterations, colorectal carcinogenesis is associated with metabolic changes which result in large numbers of serum or fecal metabolites [15]. One of these metabolites, glutamine, was present at low levels in the serum of CRC patients compared with the levels in healthy controls [12,13,14,29]. Limited studies have evaluated whether these low serum glutamine levels have a metabolic signature and prognostic significance in CRC patients. Recently, Päivi et al. revealed low serum glutamine levels were associated with poor cancer-specific survival and OS in univariate analysis, but no prognostic significance of glutamine levels was observed in multivariate analysis. In our study, patients with low glutamine levels demonstrated poorer OS and PFS than those with high glutamine levels in both univariate and multivariate analyses. As well as stage, pretreatment glutamine level was discovered using multivariate analysis to be an independent prognostic factor. Our results provide insight into the prognostic value of pretreatment serum glutamine levels in patients with CRC.

Glutamine is an important nutrient for cancer cell metabolism [30]. Several mechanisms have been proposed regarding glutamine metabolism in CRC, such as glutamine addiction, increased glutaminolysis, and autophagy activation for cancer cell survival and proliferation [31,32,33]. We speculated that the consumption of glutamine by CRC cells varies in every patient with different mutations, which may produce disparate levels of serum glutamine [33,34,35]. Serum glutamine level is maintained in circulation at a relatively constant level of 0.6–0.9 mmol/L in the normal population [17]. The decreased concentration of serum glutamine in cancer patients may be associated with higher demand by proliferating cancer cells [21]. Regardless of the diversified postulated mechanisms, our study showed that low pretreatment serum glutamine level was an independent poor prognostic factor in CRC patients. In recent decades, several trials have shown that glutamine supplementation in CRC patients may reduce chemotherapy-related side effects, such as mucositis, intestinal toxicity, and neuropathy [36,37,38]. Two large prospective studies also indicated that dietary glutamine or glutamic acid intake may reduce CRC incidence in non-overweight individuals, as well as cancer mortality [39,40]. Targeting of glutamine metabolism has recently emerged as a cancer treatment [20]. Whether glutamine supplementation to maintain adequate serum glutamine level and block glutamine intake by cancer cells can improve the outcome of CRC patients requires further studies.

Meanwhile, our research revealed decreased serum glutamine level was correlated with elevated levels of proinflammatory cytokines, which was compatible with the findings in a previous study [22]. Possible secondary consequences of persistent systemic inflammation are CRC progression and increased disease severity. Proinflammatory cytokine levels which include those of TNF-α, IL-1β, and IL-6 are commonly elevated in CRC patients [41]. These cytokines are secreted by tumor cells and cells recruited to the microenvironment. They act in oncogenic signaling pathways, such as NF-κB and STAT3, which enhance tumor survival and proliferation [42]. Our previous study demonstrated that pretreatment levels of serum TNF-α, IL-1β, and IL-6 could predict progression in CRC patients [43]. Both inversely correlated features of glutamine and proinflammatory cytokines may reflect tumor growth and survival and predict prognosis in CRC patients. 

Our study has several limitations. We enrolled only a small sample and from only one medical center; therefore, the significance of the effects of the clinical variables on survival may have been limited. In addition, our study was retrospective, and incomplete data led to some patients being excluded, which may have confounded the study results. Further prospective studies are required to validate our findings. Despite these limitations, we have demonstrated that serum glutamine level before treatment was significantly associated with the prognosis of CRC patients.

## 5. Conclusions

In summary, pretreatment serum glutamine level represents a useful and promising biomarker for prognostic evaluation in CRC patients. Future studies should be conducted to replicate our findings and investigate potential therapeutic target of glutamine metabolism to improve survival outcome in CRC patients.

## Figures and Tables

**Table 1:** Correlation of pretreatment serum glutamine levels with clinicopathological characteristics of CRC patients.

Variables	Total (*n* = 123)	Low Glutamine (<52 ng/μL, *n* = 72)		High Glutamine (≥52 ng/μL, *n* = 51)		*p*-Value
	*n*	*n*	%	*n*	%	
**Age (years)**						<0.001
<65	52	21	29.20%	31	60.80%	
≥65	71	51	70.80%	20	39.20%	
**Sex**						0.077
Male	78	41	56.90%	37	72.50%	
Female	45	31	43.10%	14	27.50%	
**BMI (kg/m**^**2**^)						0.75
<18.5	14	10	13.90%	4	7.80%	
18.5–23.9	61	35	48.60%	26	51.00%	
24–26.9	31	18	25.00%	13	25.50%	
≥27	17-	9	12.50%	8	15.70%	
**Stage**						0.019
I	27	12	16.70%	15	29.40%	
II	33	17	23.60%	16	31.40%	
III	41	24	33.30%	17	33.30%	
IV	22	19	26.40%	3	5.90%	
**Location**						0.871
Colon	83	49	68.10%	34	66.70%	
Rectum	40	23	31.90%	17	33.30%	
**Differentiation**						0.804
Well	39	22	30.60%	17	33.30%	
Moderate	76	46	63.90%	30	58.80%	
Poor	8	4	5.60%	4	7.80%	
**CEA (ng/mL)**						0.001
<5	75	35	48.60%	40	78.40%	
≥5	48	37	51.40%	11	21.60%	
**Albumin (g/dL)**						0.005
<3.5	47	35	48.60%	12	23.50%	
≥3.5	76	37	51.40%	39	76.50%	
**CRP (mg/L)**						0.003
<5	55	24	33.30%	31	60.80%	
≥5	68	48	66.70%	20	39.20%	
**mGPS**						0.004
0	72	36	50.00%	36	70.60%	
1	17	8	11.10%	9	17.60%	
2	34	28	38.90%	6	11.80%	

Abbreviations: BMI, body mass index; CEA, carcinoembryonic antigen; CRP, C-reactive protein; mGPS, modified Glasgow prognostic score.

**Table 2:** Prognostic factors for OS of CRC patients identified by univariate and multivariate Cox regression analyses.

Variables	Univariate Cox Regression			Multivariate Cox Regression		
	Hazard Ratio	95% CI	*p*-Value	Hazard Ratio	95% CI	*p*-Value
**Age** (<65 vs. ≥65 years)	1.878	0.928–3.801	0.080			
**Sex** (male vs. female)	0.807	0.419–1.557	0.523			
**BMI** (<18.5 vs. ≥18.5 kg/m^2^)	0.368	0.161–0.841	0.018			
**BMI** (<24 vs. ≥24 kg/m^2^)	0.754	0.384–1.482	0.413			
**Stage** (I vs. II vs. III vs. IV)	4.478	2.797–7.170	<0.001	3.803	2.382–6.073	<0.001
**Location** (colon vs. rectum)	1.681	0.871–3.242	0.122			
**Differentiation** (well vs. intermediate vs. poor)	1.235	0.679–2.246	0.489			
**CEA** (<5 vs. ≥5 ng/mL)	6.805	3.200–14.469	<0.001			
**Albumin**(<3.5 vs. ≥3.5 g/dL)	0.323	0.167–0.623	0.001	0.401	0.204–0.786	0.008
**CRP** (<5 vs. ≥5 mg/dL)	6.707	2.609–17.239	<0.001			
**mGPS** (0 vs. 1 vs. 2)	2.083	1.459–2.974	<0.001			
**Glutamine** (<52 vs. ≥52 ng/μL)	0.135	0.048–0.382	<0.001	0.270	0.093–0.787	0.016

Abbreviations: BMI, body mass index; CEA, carcinoembryonic antigen; CRP, C-reactive protein; mGPS, modified Glasgow prognostic score.

**Table 3:** Prognostic factors for PFS of CRC patients identified by univariate and multivariate Cox regression analyses.

Variables	Univariate Cox Regression			Multivariate Cox Regression		
	Hazard Ratio	95% CI	*p*-Value	Hazard Ratio	95% CI	*p*-Value
**Age** (<65 vs. ≥65 years)	1.490	0.742–2.995	0.263			
**Sex** (male vs. female)	0.799	0.406–1.572	0.517			
**BMI** (<18.5 vs. ≥18.5 kg/m^2^)	0.382	0.158–0.922	0.032	0.231	0.085-0.631	0.004
**BMI** (<24 vs. ≥24 kg/m^2^)	0.976	0.496–1.919	0.943			
**Stage** (I vs. II vs. III vs. IV)	7.631	4.310–13.511	<0.001	7.305	3.996-13.355	<0.001
**Location** (colon vs. rectum)	1.867	0.954–3.651	0.068			
**Differentiation** (well vs. intermediate vs. poor)	1.721	0.930–3.187	0.084			
**CEA** (<5 vs. ≥5 ng/mL)	4.746	2.319–9.716	<0.001			
**Albumin** (<3.5 vs. ≥3.5 g/dL)	0.454	0.234–0.882	0.020			
**CRP** (<5 vs. ≥5 mg/dL)	4.213	1.837–9.662	0.001			
**mGPS** (0 vs. 1 vs. 2)	1.689	1.179–2.419	0.004			
**Glutamine** (<52 vs. ≥52 ng/μL)	0.234	0.097–0.564	0.001	0.367	0.143–0.941	0.037

Abbreviations: BMI, body mass index; CEA, carcinoembryonic antigen; CRP, C-reactive protein; mGPS, modified Glasgow prognostic score.

**Table 4:** Associations of pretreatment serum glutamine and pro-inflammatory cytokines levels in CRC patients.

Cytokines (pg/mL)	Low Glutamine (<52 ng/μL, *n* = 72)	High Glutamine (≥52 ng/μL, *n* = 51)	*p*-Value
TNF-α	92.9 ± 112.3	66.7 ± 24.9	0.104
IL-1β	26.2 ± 46.3	14.0 ± 5.5	0.030
IL-6	16.4 ± 35.8	7.5 ± 4.3	0.041

Abbreviations: TNF-α, tumor necrosis factor-α; IL-1β, interleukin-1β; IL-6, interleukin-6.
